# Structural Validity and Internal Consistency of the Professional Nurse Self-Assessment A-Scale (II) for Measuring Clinical Competence Among Graduating Nordic Nursing Students

**DOI:** 10.1155/nrp/8312620

**Published:** 2025-09-30

**Authors:** Lotta Eronen, Camilla Strandell-Laine, Sigrid Wangensteen, Auvo Rauhala, Lisbeth Fagerström

**Affiliations:** ^1^Department of Health Sciences, Faculty of Science and Engineering, Åbo Akademi University, Vaasa, Finland; ^2^School of Business and Healthcare, Arcada University of Applied Sciences, Helsinki, Finland; ^3^Department of Nursing, Lovisenberg Diaconal University College, Oslo, Norway; ^4^Department of Nursing Science, Faculty of Medicine, University of Turku, Turku, Finland; ^5^Department of Health and Welfare, Novia University of Applied Sciences, Turku, Finland; ^6^Department of Health Sciences, Faculty of Medicine and Health Sciences, Norwegian University of Science and Technology, Gjøvik, Norway

**Keywords:** clinical competence, Nordic nursing students, Professional Nurse Self-Assessment A-Scale (II), structural validity and internal consistency

## Abstract

**Aim:**

To test the structural validity and internal consistency of one (A-scale) of the two Professional Nurse Self-Assessment Scale II (ProffNurse SAS II) scales among graduating Nordic nursing students with the goal of assessing their psychometric properties when used separately.

**Design:**

This study employed an explorative factor analyses to validate the ProffNurse SAS II A-scale items (*n* = 50) (self-assessed clinical competence).

**Methods:**

The data were analyzed using jamovi Version 2.3.21. The suitability of the data for the planned analyses was assessed using established criteria for good model fit prior to conducting statistical tests. Exploratory factor analysis (EFA) was used to examine the underlying structure of one of the two scales, given its application in a new population and the lack of prior confirmation of its factor structure within this specific context. Internal consistency was assessed using Cronbach's alpha.

**Data Sources:**

Convenience sampling was used to select the participants among graduating nursing students in five Nordic countries. Data collection was made digitally during 2021–2022. A total of 274 graduating nursing students from five Nordic countries and 12 different universities completed the ProffNurse SAS II A-scale holding 50 items in local languages.

**Results:**

EFA and parallel analysis elicited a five-factor solution accounting for 52.14% of variance in scores. Internal consistency reliability was supported with Cronbach's alpha of 0.960 for the ProffNurse SAS II A-scale. The five factors were named *Direct Clinical Practice (1), Ethical and Collaborative Practice (2); Pharmaceutical Practice (3); Digital Coaching and Guidance (4); and Development and Leadership (5).* The A-scale with a reduced number of items (from 50 to 44 items) showed proper structural validity and internal consistency for measuring self-assessed clinical competence and needs for further training among graduating nursing students.

**Conclusions:**

The scale supports the study's theoretical framework and competencies of the graduating nursing students.

**Implications for the Profession and/or Patient Care:**

Self-assessment supports evidence-based practice and nursing education, and the ProffNurse SAS II A-scale offers a valid tool for measuring clinical competence among graduating students.

**Reporting Method:**

The STROBE checklist for cross-sectional studies was adhered to, as well as the COSMIN reporting guideline.

**Patient or Public Contribution:**

Graduating nursing students from five Nordic countries contributed to the study by answering the questionnaire.

**Summary**

• What does this paper contribute to the wider global clinical community?
◦ This paper introduces the use of the ProffNurse SAS A-scale with reduced items (50–44), as an instrument for assessing self-assessed clinical competence, now applicable to graduating bachelor's level nursing students.
◦ Unlike existing tools, this instrument provides a comprehensive holistic approach to measuring, specifically clinical competence among nurses.
◦ It can be used by educational institutions to enhance curricula and by healthcare organizations and clinical teachers to assess clinical competence, identify training needs, and support nurses' professional development to meet evolving healthcare demands.

## 1. Introduction

Registered nurses' scope of practice can be defined as the services that nurses are competent and authorized to carry out when nursing knowledge, expertise, and compassion are needed in patient care to achieve the best outcomes for patients [1]. Registered nurses work in interprofessional healthcare teams as authorized members focusing on their specific scope of practice [1, 2]. Nursing competence is a key concept which can be defined as the integrated knowledge, skills, judgment, and attributes required of a nurse to practice safely and ethically in a designated role and setting [2]. Clinical competence, meanwhile, refers to the knowledge, skills, personal values, and ethics which are demonstrated through performance and linked to context and external factors [3, 4], meaning that general nursing competence is about overall professional ethics, responsibility, and tasks, while clinical competence is more related to specific concrete care situations.

Defining and evaluating nurses' clinical competence is complex due to the multifaceted nature of nursing practice [5, 6]. As both the demands on healthcare and the system itself are constantly evolving and changing to meet future requirements, so will nursing practice and competences [1, 7–9]. It is not just a question of a one-off change but of getting used to the fact that nursing is a profession that adapts to an ever-changing environment. Even though core nursing activities such as promoting health, alleviating suffering, contributing to a dignified death, and providing holistic person-centered care are constant [10], the nature of nursing activities evolves [8, 11]. The WHO also addresses the quality of nursing education in words like “ready to practice?” in the State of Worlds Nursing 2025 report [12]. Nursing is in a period where we constantly need to evaluate how the knowledge and clinical competence acquired by nursing students during their education correspond to and are relevant to patient safety and the needs of future nursing care, working life, and society [8, 13], as well as how continuous education can be measured to correspond to the needs of clinical practice.

Inconsistencies in assessment methods and tools across countries and institutions highlight the need to establish consistent and systematic approaches and to employ reliable, valid instruments [14]. Previously several instruments for assessing clinical competence have been deemed to have questionable or inadequate content validity, underscoring the need for further validation [15]. Therefore, continuous research on instruments for assessing clinical competence among nurses is needed. An instrument for assessing competence is an important tool for nursing education, and clinical practice [16] specifically when focusing on clinical competence and thereby offering a standardized approach to evaluating students' skills, knowledge, and professional performance, so-called “hands-on” competence [17]. There are limited tools for the specific purpose of evaluating clinical competence, and the availability of a well-validated instrument supports objective assessment and contributes to the overall quality of evidence-based nursing education and practice [16].

## 2. Background

In order to ensure patient satisfaction and the functioning of the health system from a global perspective adequate and motivated nurses are needed, and improvements are necessary to support nurses, especially new graduates on their career pathways and increase retainment in the profession [13, 18, 19]. Newly graduated nurses' satisfaction with their nursing education has been found to be statistically significantly associated with their job satisfaction, that is, satisfaction with their current job, the quality of care, and the nursing profession [10]. At the government level, research has proposed exploring and discussing how nursing education can be harmonized across regions and how to adapt curricula and specialization programs to current and future needs, for example, emphasizing health technology and the development of frameworks such as scope of practice [13, 20]. The Nordic nursing labor market is characterized by a high degree of mobility, with mutual recognition of qualifications enabling nurses to work across borders [21–23]. Nurses in the Nordic countries generally enjoy strong professional autonomy and are equal members of interdisciplinary healthcare teams. The role of educational institutions as contributors to nursing competence and as collaborators in achieving workforce goals is essential [7, 13]. Measuring the quality of education and the student's clinical competence can therefore be seen as a central part of quality assurance.

A well-validated instrument for mapping clinical competence is valuable for the development of the nursing profession, nurse education, and specialization studies [16, 24] when combined with other methods. Several instruments for assessing nurses' competence have been developed over the past decade also in within the Nordic countries, for example, the Nurse Professional Competence Scale (NPC) [15, 24], the Nurse Competence Scale (NCS) [25], and the for example ambulance NCS [26], which is focused on a particular field. Yet, instruments available for measuring specifically clinical competence are limited. A scale that is measuring clinical competence might be more beneficial for clinical practice than a scale focusing solely on general nursing competence. Overall, selecting an appropriate scale can be complicated, and the context and purpose need to be carefully considered [15]. An integrated holistic view of competence or a combination of quantitative and qualitative methods can reduce the risk of merely evaluating tasks and skills when evaluating nurses' clinical competence [4]. Therefore, a holistic approach to assessment of clinical competence has been promoted lately within the nursing profession [27]. In addition, psychometric testing of an instrument is highly important in order to ensure the results collected are valid and reliable [18]. As assessment of education should be both reliable and valid, mapping using well-tested instruments is an important part of this [19].

The Professional Nurse Self-Assessment Scale II (ProffNurse SAS II) is a questionnaire designed to specifically measure self-assessed clinical competence among nurses on a A- and B-scale of 1–10 [20–22, 28]. The A-scale measures self-assessed clinical competence and the B-scale self-assessed need for further training/knowledge on the same item. The ProffNurse SAS II holds a strong theoretical foundation as the Caring Advanced Practice Nursing Model [29] serves as a theoretical framework, giving the instrument a deductive approach [18]. The Caring Advanced Practice Nursing Model is based on the International Council of Nurses' (ICN) [30] and Hamric's definitions [31] of the central competence domains of Advanced Practice Nursing and a three-dimensional view of knowledge [15, 24]. The model defines knowledge as consisting of “epistêmê” or theoretical-scientific knowledge, “technê” or practical knowledge, and “phronesis” or wisdom [32]. The three-dimensional approach to knowledge originally stems from Aristotle's philosophy [33]. Leading to an approach where nurses' clinical skills are viewed as an intertwining of these three different types of “knowledge,” visible in the nurses' clinical competence and practice. The model emphasizes the equality of the different types of knowledge (theoretical-scientific knowledge, practical knowledge, and wisdom) in an evidence- and knowledge-based clinical care reality focused on providing high quality care. Concepts of the Nordic theory of caritative caring [29, 34] are grounded in the model and the caring communion plays a central role in the Caring Advanced Practice Nursing Model [19]. Concepts from the model's theoretical perspectives serve as philosophical approaches which take a holistic view of the patient and care and have, therefore, been integrated into the ProffNurse SAS II instrument [12]. In addition, the model identifies eight core competences in Advanced Practice Nursing: *Direct Clinical Practice, Ethical Decision-Making, Coaching and Guidance, Consultation, Collaboration, Case Management, Research and Development, and Leadership* [19]. Yet, the core competences or domains of the model are adaptable to all levels of nursing (Basic, Specialized, and Advanced Nursing), based on the idea that the same care activities are performed at different levels, with the advanced level being characterized only by deeper and broader caregiver competence within the specific area [19, 28].

ProffNurse SAS II has not yet been tested on graduating bachelor-level nursing students and may benefit from shortening to increase response rates. Additionally, a moderate negative correlation has been found between the A- (self-assessed clinical competence) and B-scale (need for further training/knowledge), meaning that low clinical competence correlates with higher need for further training/knowledge on an item and vice versa (Eronen et al. submitted). Therefore, structural validity and internal consistency was tested specifically for the A-scale.

## 3. Aim

The study aimed to test the structural validity and internal consistency of one 50-item (A-scale) of the two ProffNurse SAS II scales among graduating Nordic nursing students.

## 4. Methods

### 4.1. Design, Setting, and Sampling

The data for the study came from a cross-sectional study [35] and was collected digitally between the years 2021 and 2022 from graduating bachelor level nursing students in five Nordic countries and 12 different universities. The data were collected using a convenience sampling method, twice a year, at the time of graduation, resulting in a total of four data collections. Only students at bachelor's level were included. At the national level, one member of a Nordic research group (a Nordplus funded project) from each country was responsible for obtaining research permission, student recruitment, and data collection. The invitation letter and the anonymous questionnaire link were published on a learning platform in Norway and sent digitally to graduating students via their student email, through a contact person, or directly in other countries.

A total of 2869 nursing students who were graduating from different universities, university colleges, or universities of applied sciences in Denmark, Finland, Iceland, Norway, or Sweden were invited to participate in the study. Of the 839 students that responded, 339 answered only the background questions, and the remaining 500 answered at least some of the questions on the ProffNurse SAS II scales. A total of 274 students from 12 different universities had completed the A-scale with no missing items. A larger group consisting of 326 questionnaires (including an additional 52 incomplete questionnaires) was also analyzed at the beginning of the process for comparison. The missing A-scale items in these incomplete questionnaires were shown to be missing completely at random (MCAR). All A-scale item means of the 274 complete and the 52 incomplete answers showed no statistically significant differences between groups. As the results were quite similar, it was decided to use only the responses with no missing items (*n* = 274) for the statistical tests. Answers to B-scale items were omitted.

### 4.2. The Instrument

The ProffNurse SAS II questionnaire is a further developed and modified version of the previously validated ProffNurse SAS I questionnaire [36–39]. The instrument contains 6 components: Direct Clinical Practice, Professional Development, Ethical Decision-making, Clinical Leadership and Cooperation and Consultation, and Critical Thinking. The 50 items included measures both self-assessed clinical competence (A-scale) and the need for further training (B-scale) on two scales among nurses. The instrument is intended to be used at different educational levels, as it holds a holistic approach and addresses core clinical competencies/domains [29]. The instrument asks for (a) self-assessment of clinical competence on a scale of 1–10 (A-scale), where 1 indicates low and 10 high competence, and (b) needs for further training (B-scale), where 1 indicates low and 10 high need for further training on each item [21, 22].

The ProffNurse SAS instrument has previously been evaluated for content validity and reliability (Cronbach's alpha values ranged from 0.737 to 0.940) [21, 28], and the ProffNurse SAS II has been assessed for internal consistency, obtaining Cronbach's alpha values of 0.936 for the A-scale and 0.979 for the B-scale [37], and translated from Norwegian into Swedish, Finnish-Swedish, Finnish, Danish, Icelandic, and English [40]. The previously conducted translation process have been carried out over several stages and involved linguistic specialists, proofreading, blinded back translation, pilot testing, comparisons, and adjustments based on expert assessment.

The instrument thus consists of two scales, 50 items each and moderate negative correlations (e.g., *r* = −0.34 and *p*=0.001) between the A-scale and the B-scale have been found in previous studies that used the ProffNurse SAS questionnaire with different samples [37, 41]. A further cross-sectional study (Eronen et al. submitted) also found a moderate negative correlation (*r* = −0.47). In the latter study, 8 of the 10 items with the highest mean scores for self-assessment of clinical competence had the lowest mean scores for need for further training. Similarly, eight of the items with the highest mean scores for need for further training were also among the 10 items with the lowest mean scores for self-assessment of clinical competence. This pattern indicates that the students provided a fairly reliable evaluation of their self-assessed clinical competence and need for additional training. Based on previous findings, the questionnaire is undergoing psychometric tests only for the A-scale, with the goal of finding out whether instrument is possible to be used and further developed by retaining only the A-scale (self-assessment of clinical competence). There have been problems with the response rate and in future studies a shorter questionnaire could result in an increased response rate, as shorter versions come with several benefits [42].

### 4.3. Data Analysis

Data were analyzed statistically using jamovi (Version 2.3.21) [43] software. Little's MCAR test was performed with the R program [44], using the “naniar” package. To evaluate the structural validity and internal relationships of a scale when no clear hypothesis exists about underlying dimensions exploratory factor analysis (EFA) can be conducted [45]. As the aim was to examine the underlying structure of one of the two scales in a new population EFA was deemed suitable, instead of proceeding to confirmatory factor analysis (CFA) [46].

While EFA does not have the same formal model fit indices as CFA, there are still several statistical criteria (Kaiser–Meyer–Olkin (KMO), Bartlett's test of sphericity, factor loadings, eigenvalues, scree plot, and parallel analyses) to evaluate the adequacy of the factor solution and good model fit that can be adhered to [47]. Established model fit criteria were applied to assess the suitability of the data prior to performing statistical analyses [48]. Data analysis was conducted following the steps: assessment of suitability of the data, factor extraction and factor rotation, and interpretation. The KMO index of sampling adequacy and Bartlett's test of sphericity were used to confirm that the data were suitable for factor analysis [49, 50]. Principal axis was used as the extraction method and parallel analysis was utilized to assist in the decision concerning the number of factors to retain and compared with a scree test [50, 51]. The assumption was that the factors could correlate with each other, which is common in psychology and social sciences, for example. Therefore, instead of orthogonal rotation methods, the oblique rotation method Oblimin was chosen, which allows for the correlation of factors to be taken into account, when conducting EFA [42, 50, 52]. The factors were indeed found to correlate with each other, with a median correlation of 0.33, which supported the choice made. An adequate sample size was ensured by requiring at least five participants per item [46]. Cronbach's alpha values were used to measure the internal consistency both of the instrument as a whole and of each of its constructs, and the acceptable level of reliability was set at ≥ 0.7 [53].

### 4.4. Ethical Considerations

The study was carried out in accordance with relevant ethical guidelines and regulations [54–56]. Research permission was obtained from all participating universities in accordance with their policies by members of the research group in each of the included countries. The data were collected in collaboration with a Nordic research project that was approved by the Norwegian Center for Research Data (NSD; Norsk senter for forskningsdata/Nr. 803827). An ethical review was not required, as no sensitive data were collected, the respondents were adults, and the data received from the project did not include any confidential information. The respondents were informed about voluntary participation and anonymity in a cover letter included in the e-mail with the web-based questionnaire. When feasible, students were provided with verbal information about the study, considering the restrictions imposed by the COVID-19 pandemic. By answering the questionnaire, the respondents gave their consent to participate. Respondents were assured that participation in or withdrawal from the study would not affect their grades, as they were about to graduate and had completed their studies and participation was via an electronic questionnaire.

## 5. Results

### 5.1. Construct Validity

The construct validity of the ProffNurse SAS II A-scale was examined using EFA. The data were found to be suitable for analysis (KMO = 0.940, Bartlett's test *X*^2^ = 7571, and df = 946 (*p* < 0.001)). At first, the parallel analysis yielded a four-factor model, but after considering the theoretical background of the questions and analyzing the factor loadings, a five-factor model was considered the most accurate. Furthermore, the number of factors within ±1 range of the estimate can be considered as viable candidates [51]. The five-factor model included digital competence as a unique factor, which was not included in the four-factor model, after considering theoretical perspectives as well as statistical analysis. Additionally, four items that were classified as specific to Advanced Practice Nursing (items nos. 28, 31, 32, and 39) would have been dropped in the four-factor model. One example of an item concerning Advanced Practice Nursing which was considered important to retain is item no. 39, *I am cognizant of when my medical knowledge is insufficient when assessing patients' health conditions*.

Items that were identified as weak (factor loading < 0.4) were investigated and accurately considered from a theoretical perspective, before deleting and continuing the analysis [36, 42]. Based on the three EFAs conducted the research group chose to retain items with a factor loading of ≥ 0.35. In the first EFA, all 50 items in the ProffNurse SAS II A-scale were included. The second EFA was performed with the 48 items which remained after the first EFA. The third EFA was performed with the 44 remaining items.

The first factor, *Direct Clinical Practice,* explained 16.41% of the total variance (eigenvalue = 7.22). The second factor, *Ethical and Collaborative Practice,* explained 17.80% of the total variance (eigenvalue = 7.83). The third factor, *Pharmaceutical Practice,* explained 4.17% of the total variance (eigenvalue = 1.83). The fourth factor, *Digital Coaching and Guidance*, explained 5.45% of the total variance (eigenvalue = 2.40). The fifth factor, *Development and Leadership,* explained 8.31% of the total variance (eigenvalue = 3.66). The five factors accounted for 52.14% of the total variance. To interpret the theoretical substance of the factors the variables that loaded the strongest on each of the factors were checked and compared with the theoretical perspectives, the clinical core competencies that form the basis of the Caring Advanced Practice Nursing Model, which serves as the theoretical framework [29].

### 5.2. Internal Consistency Reliability

The internal consistency of the ProffNurse SAS II A-scale was checked using Cronbach's alpha (*n* = 274, A-scale, no missing items). The alpha value of the A-scale was 0.960 and the alpha values of the five factors ranged from 0.841 to 0.937. The alpha value for the first factor (*Direct Clinical Practice*) was 0.932 (13 items), for the second factor (*Ethical and Collaborative Practice*) 0.937 (22 items), for the third factor (*Pharmaceutical Practice*) 0.843 (2 items), for the fourth factor (*Digital Coaching and Guidance*) 0.884 (2 items), and for the fifth factor (*Development and Leadership*) 0.841 (5 items).

The results of the EFA of the ProffNurse SAS II A-scale are presented in detail per factor and per item in Table 1. The excluded items are presented in Table 2.

## 6. Discussion

The aim of the study was to test the structural validity and internal consistency of the ProffNurse SAS II A-scale among graduating (Bc) Nordic nursing students (*n* = 274) and to possibly omit the B-scale (the need for further training). EFA, using parallel analysis, identified five underlying factors in the data. The reliability of the internal consistency was proven by Cronbach's alpha which was 0.96 for the ProffNurse SAS II A-scale, which exceeds the limit for clinical reliability for instruments. Thus, the A-scale with a reduced number of items (from 50 to 44 items) showed proper structural validity and internal consistency based on the results of the statistical analysis.

### 6.1. EFA

The use of EFA was central for evaluating the structural validity of the scale within a new sample context. Given that only one of the two original subscales (the A-scale) was employed, and the sample differed demographically from those in previous studies, it was important not to assume the original factor structure would replicate. The importance of re-examining psychometric properties when changing a questionnaire or applying it to new populations is, therefore, justified. This approach allowed for a data-driven evaluation of item performance and factor structure without imposing prior constraints, ensuring the scale's appropriateness and interpretability within the new sample among graduating nursing students. EFA acts as a necessary step to examine structural validity and internal consistency before confirming or applying the one-scale version more broadly [57]. As EFAs are to be used to discover factor structure and explore underlying structures at a specific dataset without any hypothesis or prior models [47].

The five factors capture over half of the patterns or information found in participants' responses, which is considered acceptable [58]. As factor analysis attempts to identify the set of factors that represent the underlying relationship among a group of related variables the variables that had the strongest loading on each factor were considered when interpreting the theoretical substance [46].

The number of items on the ProffNurse SAS II A-scale was reduced from 50 to 44 based on factor loadings (≥ 0.35), taking into account the current needs of the healthcare setting [59] (e.g., caring for patients in a digital environment) and theoretical consideration by the research group. Further studies conducting CFAs are needed in the future to confirm the theoretical basis and the validity and reliability of the developed version in broader contexts.

### 6.2. ProffNurse SAS II A-Scale as a Clinically Reliable Instrument

Internal consistency can be defined as the interrelatedness among items [60]. The internal consistency of the factors, as measured by Cronbach's alpha, ranged from 0.84 to 0.94, thus exceeding the commonly accepted thresholds for clinical instruments. Values above 0.70 are acceptable for new scales, > 0.80 for well-established instruments, and > 0.90 indicate good internal consistency reliability [42, 53]. The factors with the most items, Direct Clinical Practice and Ethical and Collaborative Practice, achieved alpha values above 0.90. Factors with fewer items showed slightly lower alpha values (> 0.80), suggesting acceptable reliability but with potential for improvement through item revision or expansion in future studies.

### 6.3. A-Scale as the “Only” Scale

As such, the A-scale (A-scale = self-assessed clinical competence, where 1 indicates low and 10 high competence in each item) of the ProffNurse SAS II instrument shows potential to be used separately from the B-scale (B-scale = need for further training) to also investigate need for further training. As low self-assessed clinical competence seems to indicate a need for further training or knowledge within the same area [35]. There are several potential benefits to using the developed version containing only the A-scale, for example, a markedly shorter questionnaire which may lead to higher response rates [42] in future studies. However, it is important that no key items are left out given the theoretical background and competences that may be considered relevant for, for example, digitalization [11, 59] and pharmacological knowledge, where a gap have been found in previous studies [35, 37, 39, 41].

### 6.4. Theoretical Perspectives and the Five-Factor Solution

EFA confirmed the five-factor solution as the most appropriate based on theoretical perspectives and consensus among the research group. The five-factor solution is supported by the Caring Advanced Practice Nursing Model [29] and philosophical approaches involving a holistic view of the patient and care [10]. The model identifies eight core competences in Advanced Practice Nursing: *Direct Clinical Practice, Ethical Decision-Making, Coaching and Guidance, Consultation, Collaboration, Case Management, Research and Development, and Leadership.* The ProffNurse SAS II is designed to specifically measure self-assessed clinical competence from a holistic perspective among nurses at different educational levels. The theoretical foundation of the instrument is based on clinical core competences, and therefore it is deemed suitable to be used at different educational levels including advanced level [38]. These core competences arising from the theoretical foundation were shown to be appropriate to describe the factors. As such, when naming factors both the items that loaded strongest (≥ 0.35) and those which were supported by scientific theory were used.

The five-factor solution was considered important also as it retained four more items that were significant for the role of advanced practice nurse than the four-factor solution. Leaving those items out (items nos. 28, 31, 32, and 39) could have restricted the use of the instrument to graduating nursing students, which was not the purpose. Choosing the five-factor model makes the instrument more adaptable to different educational levels e.g. basic, specialized, and advanced level professional roles. As the intention is not to develop the instrument so it will be restricted to a certain setting (e.g., graduating nursing students), but to confirm its reliability and validity for various settings, educational levels, and professional nursing roles beyond the previously applied settings, and to potentially shorten the questionnaire [37–39].

One new factor arises in the five-factor solution which can be seen important considering technological development [11, 59] within the healthcare sector. As the healthcare sector is undergoing rapid developments due to factors such as technology and digitalization, nursing education is also evolving in a progressive manner [61]. Previous studies have found knowledge gaps within eHealth [35, 37, 41]. The five-factor model retained items on the factor named *Digital Coaching and Guidance*, which can be seen as particularly valuable to the present and future of the nursing profession [59, 62, 63].

### 6.5. Excluded Items

The shortened version contains 44 items instead of 50. Of the six excluded items (factor loading < 0.35), three did not load on any of the factors in the EFA (for factor loadings, see Table 2); e.g., item no. 47, *I give health promotion and illness preventive recommendations in accordance with national guidelines to patients,* might be too self-evident, as nurses are required to adhere to national guidelines in their everyday work. In addition, item no. 37, *I consult other professional experts when required*, could potentially be covered by item no. 38 on cooperation with other professionals, while item no. 35, *I experience a division of responsibility between the physician and me as a nurse,* might be unnecessary considering cooperation is asked about elsewhere (e.g., item no. 38). Item no. 13, *I develop and administer health-promoting and illness-preventive actions for patients,* was close to the loading limit (0.357) but after discussions within the research group it was decided to exclude the item due to similarities with several other questions on similar themes, for example, *I identify patients' health problems* (item no. 4) and *I take patients' physical health needs (illness, pain, disabilities, etc.) into account when assessing and planning for the health and life situation of patients (item no. 23).* The last excluded item (no. 50), *I report all incidents in accordance with the actual patient safety system*, could be considered to be covered by item no. 33, *I am correct and accurate in speech and writing*. Presumably, the exclusion of items measuring the same thing succeeded. It must be taken into account that when using self-assessment, some answers become a matter of personal interpretation also for the respondent. Excluded items are presented in Table 2.

### 6.6. Self-Assessment as a Measurement Tool

As the scale only measures clinical competence from the perspective of the nurse, this means that, in addition to personal interpretation, the bias associated with self-assessment [64, 65] must be taken into account; for example, there might be variations depending on whether the respondents are nursing students or experienced professionals, and whether they are too critical of themselves or over- or undervalue their competence [65–68]. As such the setting and sample need to be considered carefully when applying the instrument. Studies show that self-assessment is a proper and well-functioning method when participants are used to self-assessment and when it is pointed to a specific area of knowledge, for example, nursing, and not general knowledge across different areas [64, 69]. Despite some limitations, considering self-assessment as a method the instrument can easily provide valuable information on the clinical competence of nurses and their need for further training.

### 6.7. Future Research

The ProffNurse SAS II A-scale with reduced number of items (from 50 to 44) shows proper structural validity and internal consistency and may, therefore, also be applied among nursing students, in addition to the variety of nursing settings where the ProffNurse SAS II already has been applied [36, 38–40]. Using the shorter ProffNurse SAS II questionnaire would be preferable in the future considering increasing response rates. Due to the development within the profession the instrument may require updating, changes, and new items in order to measure self-assessed clinical competence as accurately as possible, specifically about digital care and competences needed to care for patients remotely and via different eHealth solutions [9, 59]. The need for revisions and the introduction of new questions might be needed quite frequently, in addition to conducting CFA.

### 6.8. Practical Relevance

Both educators and employers can benefit from the shortened version of the ProffNurse SAS II instrument, which uses a theory-based holistic approach to evaluate the development of clinical competence of nurses and to assess the relevance of education to the needs of the health sector. Moreover, the information obtained can positively impact on the career path of nurses if taken into account by leaders in healthcare organizations, clinical educators, and educational institutions, for example, when planning mentoring, further training, and specialization programs. It can also provide healthcare educators, supervisors, clinical educators, and healthcare leaders with timely information about the clinical competence of nurses, along with other methods. The ProffNurse SAS II A-scale can be completed by registered nurses, advanced practice nurses [36–39], or as part of nursing education as the theoretical basis supports the broad applicability of the instrument, considering that core clinical competences are general in nature [29].

## 7. Strengths and Limitations

### 7.1. Sampling and Generalizability

First, with regard to sampling, the sample consisted of graduating nursing students in different universities in the Nordic countries, but the number of participants from different countries was not equal, and a convenience sampling strategy was used. The Nordic countries vary in size and different numbers of institutions provide nursing education in each, for example, Iceland has only two universities and both were included, while the other countries have several and not all could be included due to time constraints. As such, the sample consisted of graduating nursing students from the Nordic countries as a region, meaning participants should not be considered fully representative of the population. Self-assessment can lead to bias, which needs to be addressed and can raise questions about the generalizability of the results.

Additionally, response rates were lower than desired, given the large number of potential responses. More representative samples from different stages of education are needed in future studies to test the psychometric properties of the ProffNurse SAS II A-scale. As the results are restricted to the Nordic region, further validations are required to support the five-factor solution of the ProffNurse SAS II A-scale in other geographic settings. It also needs to be considered that the Nordic region follows the same European Union (EU) directives and has similar education for nurses at the bachelor level. It is possible that the questionnaire and the items work differently when applied outside the EU area.

Second, there are several viewpoints regarding what constitutes an adequate sample size for conducting factor analysis. The sample size (*n* = 274, A-scale, no missing items) can be seen as a limitation, as the response rate remained low compared with the number of potential responses. Despite this, based on different viewpoints, the data used in this study seem to fulfill the requirements of 150+ with at least five cases of each variable [52, 46] considered adequate for conducting exploratory factor analyses. The 274 survey responses included in this study had no missing items, which can be seen as a strength, especially because the missing values in the other returned surveys were MCAR and no differences were found between the item means of complete and incomplete answers. Including imputed responses (*n* = 326) in the statistical analyses was also evaluated but as the results were quite similar, only responses with no missing items were retained for the psychometric testing.

### 7.2. Parallel Analysis

Parallel analysis was chosen for determining the number of factors in EFA as it is considered the most appropriate method [50–52], and the numbers of factors should range between 1 of the estimate [51]. The parallel analysis yielded a four-factor model; however, after discussion among the research group and considering theoretical perspectives, this was changed to a five-factor model, still an acceptable number, which can be seen as a strength. Third, the chosen model has two factors with only two questions, which can be considered a weakness. They were theoretically discussed and decided on to retain.

Finally, the conditions of the model chosen based on factor analysis are in principle partially contradictory to each other: the proportion of overall variance in the data that is accounted for by the solution should be as high as possible. The number of factors should be as low as possible. The model should contain as many large and small loadings of absolute value as possible. The factors should have a meaningful interpretation [45].

An important strength of the study is the adherence to the STROBE [70] checklist for cross-sectional studies and the COSMIN reporting guideline [71] (checklist as Supporting file (available here)), thereby ensuring standard of methodological accuracy, transparency, and reporting quality.

## 8. Conclusion

The ProffNurse SAS II A-scale, a shortened version of the original instrument, has shown good structural validity and internal consistency for assessing self-assessed clinical competence and training needs among graduating bachelor-level nursing students. While several tools exist to measure nursing competence, instruments specifically targeting clinical competence from a holistic, theory-based perspective remain limited. The updated ProffNurse SAS II addresses the gap and can now be used to evaluate competence development at the bachelor level. The results provide valuable information on how training matches the competence needs of clinical practice. In addition, the results contribute to the nurse's career path if they serve as a decision-making basis for healthcare leaders and clinical educators, especially when planning mentoring and specialization programs. Further research, including studies with broader, non-Nordic samples and CFA, is needed to validate the instrument's factor structure and generalizability.

## Figures and Tables

**Table 1:** Exploratory factor analysis of the ProffNurse SAS II A-scale.

Factor (number of items)	Cronbach's α	Content (item no.)	Loading	Cronbach's α if dropped
1 = Direct clinical practice (13)	**0.932**	I assess patients' symptoms (5)	0.775	0.925
		I identify patients' health problems (4)	0.740	0.925
		I evaluate and modify patients' medical treatment (6)	0.691	0.926
		I apply both subjective and objective methods when examining, treating, and caring for patients (9)	0.652	0.926
		I plan and prioritize nursing and medical interventions (3)	0.650	0.926
		I am independently responsible for health assessment (systematic physical examination), examinations, and treatment of patients with complicated medical conditions (1)	0.636	0.927
		I interpret, analyze, and reach alternative conclusions about patients' health conditions after a detailed mapping of health history and health assessment (physical examination) (8)	0.599	0.924
		I utilize medical equipment in an appropriate and accurate manner (10)	0.598	0.929
		I exclude alternative diagnoses when assessing patients' health conditions (7)	0.577	0.926
		I identify changes in patients' health and medical conditions (12)	0.564	0.926
		I am independently responsible for health assessment (systematic physical examination), examinations, and treatment of patients with uncomplicated medical conditions (2)	0.552	0.928
		I am correct and accurate in speech and writing (33)	0.550	0.931
		I make my own decisions in my work (31)	0.352	0.929

2 = Ethical and collaborative practice (22)	**0.937**	I take patients' physical health needs (illness, pain, disabilities, etc.) into account when assessing and planning for the health and life situation of patients (23)	0.660	0.934
		I take patients' mental health needs (mood swings, feelings of hopelessness, depression, etc.) into account when assessing and planning for the health and life situation of patients (21)	0.655	0.933
		I take patients' spiritual health needs (feelings of meaninglessness, existential needs, beliefs, fear of death, etc.) into account when assessing and planning for the health and life situation of patients (22)	0.648	0.934
		I act ethically when caring for patients (24)	0.648	0.934
		I put emphasis on patients' own wishes when assessing and planning for nursing care and medical treatment (30)	0.630	0.934
		I identify and take patients' own health resources into account when planning nursing care (25)	0.624	0.933
		I reflect on my actions (41)	0.568	0.935
		I have a supportive ongoing dialog with patients about their needs and wishes (48)	0.561	0.933
		I am actively responsible for my own professional development (20)	0.535	0.934
		I support and guide patients in mastering their illnesses and health problems (27)	0.529	0.933
		I cooperate actively with other health professionals when coordinating patients' nursing, care, and treatment (38)	0.504	0.933
		I document the steps taken in assessing patients' needs for nursing, care, and treatment (40)	0.495	0.934
		I take active part in creating a good working environment (29)	0.495	0.936
		I am cognizant of when my medical knowledge is insufficient when assessing patients' health conditions (39)	0.463	0.936
		I analyze and evaluate my work continuously (42)	0.461	0.935
		I understand the consequences my decisions may have for patients (34)	0.458	0.935
		I focus on relatives' need for support and guidance (49)	0.437	0.934
		I take patients' social health needs (leisure activities, friends, financial situation, etc.) into account when assessing and planning for the health and life situation of patients (26)	0.437	0.936
		I take full responsibility for my own actions (32)	0.435	0.935
		I maintain an ethical approach towards my colleagues (28)	0.401	0.959
		I perceive opportunities and have visions for how nursing and clinical paths for patients can be developed (43)	0.372	0.934
		I systematically gather information from each patient about her/his health resources (14)	0.364	0.935

3 = Pharmaceutical practice (2)	**0.843**	I have knowledge of the interactions of various types of medication and what side-effects they may cause for the patients I am responsible for (15)	0.581	0.819
		I have knowledge of the medication effects and treatment for the patients I am responsible for (11)	0.575	0.656

4 = Digital coaching and guidance (2)	**0.884**	I give health promotion advice and recommendations to patients by telephone, email, or other health technology solutions (46)	0.819	0.802
		I assess patients' health needs by telephone, email, or other health technology solutions (45)	0.807	0.781

5 = Development and leadership (5)	**0.841**	I participate in quality development at my workplace (17)	0.761	0.800
		I take responsibility for competence development at my workplace (18)	0.733	0.797
		I improve routines/systems that fail to meet the needs of patients at my workplace (19)	0.659	0.795
		I generate a creative learning environment for staff at my workplace (16)	0.571	0.817
		I have a vision of how nursing should be developed at my workplace (44)	0.495	0.834

*Note:* Extraction method: principal axis factoring. Rotation method: Oblimin. Values show the highest factor loadings. Factor structure is based on parallel analysis. Communality cutoff value 0.35. The bold values are the Cronbach's α per factor.

**Table 2:** Excluded items.

Item	Content	Factor	Highest loading	Not included due to factor loading
13	I develop and administer health-promoting and illness-preventive actions for patients	2	0.357	Theoretical consideration
35	I experience a division of responsibility between the physician and me as a nurse	—	—	< 0.35
36	I cooperate well with the physician	1	0.336	< 0.35
37	I consult other professional experts when required	—	—	< 0.35
47	I give health promotion and illness preventive recommendations in accordance with national guidelines to patients	—	—	< 0.35
50	I report all incidents in accordance with the actual patient safety system	1	0.336	< 0.35

## Data Availability

The data supporting this study are part of an ongoing doctoral dissertation and are not publicly available but will be provided upon reasonable request after its completion.

## Supporting Information

As the data for this study were collected using a cross-sectional design, the STROBE checklist was followed to ensure that all essential items for reporting cross-sectional studies were addressed. Furthermore, because the study focused on the evaluation of measurement properties, the COSMIN reporting guideline on measurement properties of PROMS was also applied to provide transparent and standardized reporting of measurement studies.
