# Supplementary material for: Structural Validity and Internal Consistency of the Professional Nurse Self-Assessment A-Scale (II) for Measuring Clinical Competence Among Graduating Nordic Nursing Students
Source: Nurs Res Pract. 2025 Sep 30;2025:8312620. doi: 10.1155/nrp/8312620 (PMC12503965; doi:10.1155/nrp/8312620)
Supplement: Supporting Information — Additional supporting information can be found online in the Supporting Information section. [file 8312620.f1.zip › Cosmin Reporting Guideline table word.docx]

| **The COSMIN reporting guideline for studies on measurement properties of PROMs - Table 1** | | | | |
| --- | --- | --- | --- | --- |
| **Report section: Title** |  | **CHECKING** | **PAGE and LINE** | **KEY STATEMENT** |
| **T1 Title** | Identify the report as a study of one or more measurement properties of a specific PROM to measure a specified construct in a specified population. | (measurement properties)✓ (a specific PROM)✓ ( a specified construct)✓ (a specified population)✓ | Title page | Structural Validity and Internal Consistency of the Professional Nurse Self-Assessment A-Scale(II) for Measuring Clinical Competence among Graduating Nordic Nursing Students |
| **Report section: Abstract** | |  |  |  |
| **A1 Objectives** | Provide the specific objective(s) of the research, specifying (1) the name (and version, if relevant), and construct(s) of the PROM, (2) the measurement properties being evaluated, and (3) relevant study characteristics. | (1 name) ✓,  (2 measurement properties) construct validity & internal consistency ✓  (3 the relevant study characteristics) ✓ | Page 2, Abstract. | ProffNurse SAS II, Self-assessment of clinical competence, Structural Validity and Internal Consistency of the A-scale among Nordic graduating (Bc) nursing students. |
| **A2 Design** | Specify (details of the) study design used to evaluate the measurement properties. | **✓** | Page 2, Line 35-37 (Abstract) | Quantitative cross-sectional design, structural validity and internal consistency |
| **A3 Methods** | Specify the methods for evaluating each measurement property. | ✓ | Page 2, Line 38-43 (Abstract) | EFA, parallel analysis and Cronbach’s alpha. |
| **A4 Results** | Provide the main results for all measurement properties evaluated. | ✓ | Page 2, Line 49-56 (Abstract) | Five-factor solution accounting for 52.14 % of variance in scores. Cronbach’s alpha of 0.960 for the ProffNurse SAS II A- scale. Reduced number of items (from 50 to 44 items). |
| **A5 Discussion/Conclusions** | Provide a brief statement of the implications of the findings in the context of existing evidence on the PROM. | ✓ | Page 3, Line 61-69 (Abstract) | Limited validated tools specifically designed to measure clinical competence. ProffNurse SAS II A-scale holding 44 items was proven to be applicable for mapping self-assessed clinical competence among graduating nursing students. |
| **Report section: Introduction** | |  |  |  |
| **I1 PROM** | Specify the name and, if relevant, the version, and construct(s) of the PROM. | ✓ | Page 6, Line 153-180 (Background) | ProffNurse SAS II A-scale. |
| **I2 Target population & context of use** | Specify the target population and context of use that the PROM was designed for. | ✓ | Page 6; Line 153-180 (Background) Page 7-8; Line 203-225 (Methods; design, setting and sampling) | Nordic graduating Bc nursing students, ProffNurse SAS is designed to specifically measure self-assessed clinical competence among nurses at different educational levels. |
| **I3 State of knowledge & Rationale** | Provide a description of the current scientific knowledge (what is known and not known) regarding the measurement properties of the PROM. Explain why the new study is necessary. Provide citations for the original development paper(s). | ✓ | Page 6-7, Line 182-188 (Background) Page 8; line 240-247 (Methods; the instrument) | ProffNurse SAS II has not yet been tested on graduating Bc nursing students and may benefit from shortening. Previously been evaluated for content validity and reliability [20, 22], and for internal consistency (32), and translated (33). |
| **I4 Objectives** | Provide the specific objective(s) of the research, specifying (1) the name (and version, if relevant) of the PROM, (2) the measurement properties being evaluated, and (3) relevant study sample characteristics. The use EFA was central for evaluating the structural validity of only the A- scale within a new sample context. | (1) ✓, (2) ✓, (3) ✓ | Page 7, Line 193-196 (The study) | Test the structural validity and internal consistency of one 50-item (A-scale) of the two ProffNurse SAS II scales among graduating (Bc) nursing students. Additionally shortening to only the A scale. |
| **Report section: General Methods** | |  |  |  |
| **GM1 Study design** | Specify (details of the) study design used to evaluate the measurement properties. | ✓ | Page 9-10, Line 265-288 (Data analysis) | Details of data analysis conducted using jamovi (version 2.3.21). |
| **GM2 Participants** | Specify how the study participants were selected. Specify the inclusion and exclusion criteria | ✓ | Page 7-8; Line 214-225 (Methods; design, setting and sampling) | Digitally, 2021 and 2022, graduating Bc nursing students in 5 Nordic countries and 12 different universities. Convenience sampling method, twice a year, at the time of graduation, a total of four data collections. Only students at bachelor’s level were included. |
| **GM3 PROM** **details** | Provide details about the original version of the PROM as well as of the PROM version being studied, specify the conceptual framework (reflective/formative model), details on the structure (the number of items and subscales), the language, response scale, recall period, direction of scoring, and scoring algorithm of the PROM. Specify how the PROM was administered (e.g., in what setting, mode of administration (e.g. paper, electronic) what instructions were given), including the country in which it is administered | ✓, Conceptual framework included in background. | Page 6; Line 153-180 (Background),  Page 7; Line 203-212 (Methods; Design, setting and sampling)  Page 8-9; Line 228-263 Methods; The instrument) (The instrument) |  |
| **GM4 Additional data collection** | Describe why and how other data was collected (e.g., construct and measurement properties of the comparator instruments, characteristics of groups being compared, and rationale for choosing groups), including mode of administration (e.g., paper, electronic). | ✓ | Page 7; Line 203-212 (Methods; Design, setting and sampling) | Digitally on a learning platform or via student email, through a contact person, or directly. At the national level, one member of a Nordic research group from each country was responsible for data collection. |
| **GM5 Time points procedures** | Provide all time points of all measurements. | ✓ | Page 7, Line 203-207 (Methods; Design, setting and sampling) | 2021 and 2022, twice a year, at the time of graduation, a total of four data collections. |
| **GM6 Justification for sample size** | Provide a rationale for the sample size for all measurement properties analyses (including subgroups). | ✓ | Page 10; Line 285-286 (Methods; Data analysis) Page 20; Line 547-551 (Strenghts and limitations; Sampling and generazilability) | An adequate sample size was ensured by requiring at least five participants per item (44). The sample size (n=274, A scale, no missing items) used in this study seem to fulfill the requirements of 150+ with at least five cases of each variable (43,44) considered adequate for conducting EFA. |
| **GM7 Statistical analyses** | Describe the statistical analyses corresponding to all objectives (see measurement properties specific boxes). Describe the criteria for good measurement properties. Name the statistical package used and the version. | ✓ | Page 9-10; Line 266-288,  Page 9-10, Line 273-288. (Data analysis) | Several statistical criteria (KMO, Bartlett’s test of sphericity, factor loadings, eigenvalues, scree plot and parallel analyses) to evaluate the adequacy of the factor solution and good model fit were adhered to. Established model fit criteria were applied to assess the suitability of the data prior to performing statistical analyses. |
| **GM8 Missing data** | Describe approaches for dealing with missing data. | ✓ | Page 7-8, Line 216-225 (Methods; Design, setting and sampling) | All A scale item means of the 274 complete and the 52 incomplete answers showed no statistically significant differences between groups. As the results were quite similar, it was decided It was decided to use only the responses with no missing items (n=274) for the statistical tests. |
| **GM9 Unplanned analysis** | Specify analyses that were unplanned and their rationale. | ✓ | Page 8, Line 219-221 (Methods; Design, setting and sampling) | Analyses comparing no missing items and answers holding incomplete answers. |
| **Report section: General results** | |  |  |  |
| **GR1 Participant characteristics** | Provide study participants’ characteristics, specified per subgroup if applicable. | ✓ | Page 7-8, Line 214-219 (Methods; Design, setting and sampling) | Denmark, Finland, Iceland, Norway, and Sweden. 274 students from 12 different universities had completed the A-scale with no missing items. |
| **GR2 Sample size** | Provide the total number of participants included in the study and the sample size for each analysis. | ✓ | Page 8, Line 218-219 (Methods; Design, setting and sampling) | n=274, data used in this study seem to fulfill the requirements of 150+ with at least five cases of each variable. |
| **GR3 Missing data** | Provide amount of (proportion or count) and reasons for missing data for each analysis for the PROM, and for any analyses of other outcome measurement instruments. | ✓ | Page 8, Line 224-225 (Methods; Design, setting and sampling) | Only the responses with no missing items (n=274) were used for the statistical tests. |
| **GR4 Results** | Describe the results corresponding to all objectives (see measurement properties specific boxes). | ✓ | Page 10-12 (Results) | EFA results presented, int.consistency: results presented. Based on the three EFAs conducted the research group chose to retain items with a factor loading of ≥0.35. The five factors accounted for 52.14% of the total variance. The alpha value of the A scale was 0.960 and the alpha values of the five factors ranged from 0.841-0.937. |
| **Report section: Discussion/conclusions** | |  |  |  |
| **DC1 Measurement property evidence** | Provide the main findings and if each measurement property is sufficient or insufficient and why. | ✓ | Page 14-15, Line; 358-365 (Discussion) | The internal consistency reliabilities as measured using Cronbach’s alpha coefficient were 0.960 for the A scale and 0.841-0.937 for the five factors separately, thus exceeding the minimum value of 0.7 for new scales, >0.80 for well-established instruments, and >0.90 for clinically reliable tools and internal consistency reliability (21,45). No. of items reduced from 50 to 44 based on factor loadings (≥0.35), taking into account the current needs of the healthcare setting (e.g., caring for patients in a digital environment) and theoretical consideration. |
| **DC2 Practical relevance** | Discuss the practical relevance of the findings in terms of recommendations for (not) using the PROM. | ✓ | Page 19, Line 492-496, 508-513 (Discussion; Future research and practical relevance) | Can be applied among nursing students. In addition to the variety of nursing settings and educational levels where the ProffNurse SAS II previously has been applied (23,25,26,33). The information obtained can positively impact on the career path of nurses if taken into account by leaders in healthcare organizations, clinical educators and educational institutions. |
| **DC3 Strengths and limitations** | Discuss strengths and limitations of each study. For example, discuss if there were any potential biases in the study that could have impacted the results. | ✓ | Page 20-21 (Strengths and limitations) | Limitations: Low response rate in comparison to possible answers, bias regarding self-assessment, generalizability (commented separately below), Strenghts: adeherence to guidelines, no missing items, analyzing method (parallel analysis), adequate sample size, theoretical foundation. |
| **DC4 Generalizability** | Discuss generalizability of the results. For example, discuss whether the results could be generalized to other populations given the sample studied. | ✓ | Page 20, Line 541-543, 530-532 (Sampling and Generalizability) | Nordic countries share an adherance to EU-directives considering Bc-level nursing education, may not be generalized outside EU-region for nursing students. |
| **DC5 Instrument changes** | Discuss what modifications are needed to the existing PROM. | ✓ | Page 19, Line 496-501, (Discussion; Future research) Page 20, Line 538-540 (Strenghts and Limitations; Sampling and generalizability) | Confirmatory Factor Analysis (CFA) is recommended in future studies to test and confirm the factor structure identified. Due to the development within the profession the instrument may require updating, changes, and new items in order to measure self-assessed clinical competence as accurately as possible, specifically with regard to digital care. |
| **DC6 Future research** | Describe new research questions or hypotheses generated from these findings, and provide/describe the research needed to answer those questions. | ✓ | Page 19, Line 495-500 (Discussion; Future research) | More representative samples outside the Nordic Region. |
| **DC7 Conclusions** | Provide the overall conclusions for the use of the PROM. | ✓ | Page 21-22 (Conclusions) | The further developed ProffNurse SAS II instrument (reduced items, only A-scale) which takes a theory-based holistic approach, can now be used to evaluate the development of clinical competence also among Bc-level nursing students. The results reflect the content of the training against the needs of the health sector. |
| **Report section: Other information** | |  |  |  |
| **O1 Conflict of interest** | State any conflict of interest you may have related to the PROM. This may include any involvement in the development of the PROM or any commercial funding or profit. | ✓ | Page 22, Line 589-592 (Funding and Conflict of Interest) | This study did not receive any public funding. The authors declare no conflict of interest. Two of the original questionnaire designers are part of the author’s team as co-writers. |
